# Host Genetic Background Influences the Response to the Opportunistic *Pseudomonas aeruginosa* Infection Altering Cell-Mediated Immunity and Bacterial Replication

**DOI:** 10.1371/journal.pone.0106873

**Published:** 2014-09-30

**Authors:** Maura De Simone, Lorenza Spagnuolo, Nicola Ivan Lorè, Giacomo Rossi, Cristina Cigana, Ida De Fino, Fuad A. Iraqi, Alessandra Bragonzi

**Affiliations:** 1 Infection and Cystic Fibrosis Unit, IRCCS San Raffaele Scientific Institute, Milano, Italy; 2 School of Biosciences and Veterinary Medicine, University of Camerino, Camerino, Italy; 3 Department of Clinical Microbiology and Immunology, Sackler Faculty of Medicine, Tel Aviv University, Tel Aviv, Israel; French National Centre for Scientific Research, France

## Abstract

*Pseudomonas aeruginosa* is a common cause of healthcare-associated infections including pneumonia, bloodstream, urinary tract, and surgical site infections. The clinical outcome of *P. aeruginosa* infections may be extremely variable among individuals at risk and patients affected by cystic fibrosis. However, risk factors for *P. aeruginosa* infection remain largely unknown. To identify and track the host factors influencing *P. aeruginosa* lung infections, inbred immunocompetent mouse strains were screened in a pneumonia model system. A/J, BALB/cJ, BALB/cAnNCrl, BALB/cByJ, C3H/HeOuJ, C57BL/6J, C57BL/6NCrl, DBA/2J, and 129S2/SvPasCRL mice were infected with *P. aeruginosa* clinical strain and monitored for body weight and mortality up to seven days. The most deviant survival phenotypes were observed for A/J, 129S2/SvPasCRL and DBA/2J showing high susceptibility while BALB/cAnNCrl and C3H/HeOuJ showing more resistance to *P. aeruginosa* infection. Next, one of the most susceptible and resistant mouse strains were characterized for their deviant clinical and immunological phenotype by scoring bacterial count, cell-mediated immunity, cytokines and chemokines profile and lung pathology in an early time course. Susceptible A/J mice showed significantly higher bacterial burden, higher cytokines and chemokines levels but lower leukocyte recruitment, particularly neutrophils, when compared to C3H/HeOuJ resistant mice. Pathologic scores showed lower inflammatory severity, reduced intraluminal and interstitial inflammation extent, bronchial and parenchymal involvement and diminished alveolar damage in the lungs of A/J when compared to C3H/HeOuJ. Our findings indicate that during an early phase of infection a prompt inflammatory response in the airways set the conditions for a non-permissive environment to *P. aeruginosa* replication and lock the spread to other organs. Host gene(s) may have a role in the reduction of cell-mediated immunity playing a critical role in the control of *P. aeruginosa* infection. These results now provide a basis for mapping genomic regions underlying host susceptibility to *P. aeruginosa* infection.

## Introduction

*P. aeruginosa* is one of the major and dreaded source of infections responsible for causing millions of cases each year in the community and 10–15% of all healthcare associated infections, with more than 300,000 cases annually in the EU, North US and Japan. Patients at risk of acquiring *P. aeruginosa* are particularly those hospitalized in intensive care units (ICU) who may develop ventilator-associated pneumonia (VAP) and sepsis [1]. In general, patients with a compromised immune system, due to immunosuppressive therapies or underlying diseases such as cancer, AIDS or the hereditary disease cystic fibrosis (CF), are at risk to develop *P. aeruginosa* infection.

The clinical outcome of *P. aeruginosa* infections may be extremely variable among individuals at risk and CF patients. In particular, heterogeneity in the severity of chronic bronchopulmonary *P. aeruginosa* infection is well documented in CF, while in other patients remains to be established [2]. According to clinical studies, the progression and severity of pulmonary disease in CF do not appear to correlate with the type of *Cystic Fibrosis Transmembrane Regulator* (*CFTR)* variant and rather seem to be largely dependent on secondary factors [3]. Much influence on disease outcome has been attributed more to different *P. aeruginosa* phenotypes rather than to host genetic background. Consistent with its larger genome size and environmental adaptability, *P. aeruginosa* contains the highest proportion of regulatory genes observed for a bacterial genome, which lead to large and complex phenotypic versatility. Thus, early studies from different groups including ours [4], [5], [6], [7] highlighted the responsibility of particular *P. aeruginosa* phenotypes for differential disease manifestations and pathogenesis. For instance, the shift from the opportunistic toward a life-long persistent phenotype has a major impact in dampening the innate immune recognition and deteriorating the lung function [8]. These studies somewhat neglected the potential importance of host factors. More recently, special interest has shifted toward understanding host genetic variation that alters the outcome of *P. aeruginosa* infection [9]. Identifying and tracking risk factors for *P. aeruginosa* infection remains one of the major research challenge.

From studies of genetic predisposition in other infectious disease it has become clear that the host response is not only influenced by single genes but by combinations of genes and their variants [10], [11]. Thus, complex (multi-gene) genetic effects need to be analyzed to understand the full repertoire of host responses to pathogens. Several candidate gene association studies have been carried out in humans. However, although studies in humans are essential, they are limited because of the size of cohorts, strong but often unknown environmental influences, poor diagnosis, and lack of repeatability [9]. Therefore, animal models are absolutely essential to complement human studies [12].

To meet the current challenge of deepening genetic susceptibility to infection and dissection of genetic traits analysis, well-defined mouse genetic reference populations (GRPs) have been a powerful force. Mouse GRPs are available as inbred laboratory and wild-derived mouse strains, recombinant inbred strains, interspecific recombinant inbred strains, chromosome substitution strains, and consomic strains [13]. More than 200 commercially available, phylogenetically diverse inbred mouse strains that contain enough genetic diversity to identify major differences in response to a specific infection are available [14]. These resources have been extensively used to identify cellular and molecular factors that may contribute to different disease pathogenesis and to analyze the effect of multiple contributing genetic loci influencing disease phenotype with different pathogens [10], [12], [15], [16] [17]. Successful stories included gene mapping for a large number of pathogens like bacteria (e.g. *Salmonella enterica serovar Typhimurium*, *Mycobacterium bovis*, *Bacillus anthracis*, *Staphilococcus aureus* and *Legionella pneumophila*), parasites (e.g. *Plasmodium chabaudi*, *Candida albicans,* and *Leishmania donovani*) and viruses (e.g *Cytomegalovirus, Vescicular stomatitis virus* and *Orthomyxovirus)* [14], [18]. Although inbred mouse strains have been used in order to describe different susceptibility to *P*. *aeruginosa* infection [19], [20], [21], nonetheless, to our knowledge mapping for genetic determinant(s) has not been reported until now.

As a first step toward the analysis of genetic traits influencing resistance and susceptibility to *P. aeruginosa* infection and the characterization of pathogenetic mechanims, we screened nine inbred mouse strains of differing ancestry and chosen for the known differences in their ability to overcome infections with various pathogens. Using characterized mouse model of acute infection with *P. aeruginosa* clinical strains and previous experience in this model system [22], [23], we identified mouse strains presenting deviant clinical and immunological phenotypes amenable for biological and genetic analyses.

## Results

### Survival and body weight of *P. aeruginosa*-infected inbred mice is strongly dependent upon genetic background

**Figure 1:**
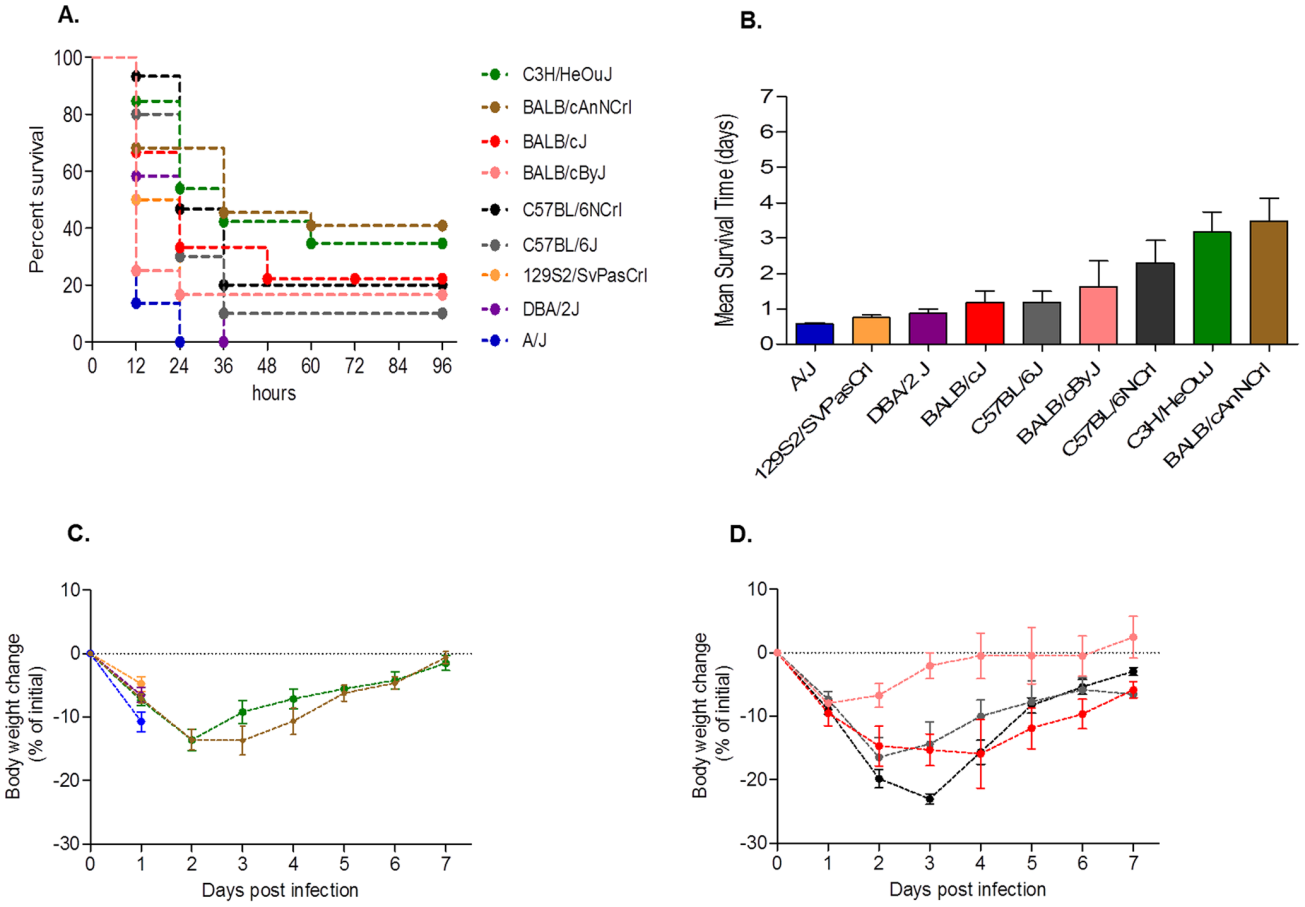
Survival, body weight and mean survival time after *P. aeruginosa* infection in inbred mouse strains. A/J (n = 22), BALB/cJ (n = 9), BALB/cAnNCrl (n = 8), BALB/cByJ (n = 12), C3H/HeOuJ (n = 26), C57BL/6J (n = 10), C57BL/6NCrl (n = 15), DBA/2J (n = 12), and 129S2/SvPasCRL (n = 12) mice were inoculated with 5×10^6^ CFU of *P. aeruginosa* clinical isolate AA2, and monitored for survival (**A**) and weight change for a period of seven days after infection (**C, D**). In addition, mean survival time was calculated based on the survival curve (**B**). Bar represent mean values and the error bars the standard error of the mean (SEM). The data are pooled from two to four independent experiments. Statistical significance by Mantel-Cox test for survival (**A**), One- way ANOVA with Bonferroni's Multiple comparison test (**B**) for mean survival time and Two-way ANOVA with Bonferroni's Multiple comparison test (**C, D**) is reported in **Table S1–S3**.

Nine different inbred mouse strains, including A/J, BALB/cJ, BALB/cAnNCrl, BALB/cByJ, C3H/HeOuJ, C57BL/6J, C57BL/6NCrl, DBA/2J, and 129S2/SvPasCRL were infected with 5×10^6^ CFU of planktonic *P. aeruginosa* clinical isolate AA2 via intra-tracheal injection, and monitored for change in body weight and mortality over a period of seven days. As shown in **Fig. 1**, a wide-range of survival and weight loss were observed among different inbred mice. The most deviant survival phenotypes were observed for A/J, 129S2/SvPasCRL and DBA/2J showing high susceptibility and BALB/cAnNCrl and C3H/HeOuJ showing more resistance to *P. aeruginosa* infection. BALB/cJ, BALB/cByJ, C57BL/6J, C57BL/6NCrl showed intermediate phenotype. In more detail, susceptible A/J, 129S2/SvPasCRL and DBA/2J died within the first two days of infection, showed a mean survival time of around one day and a rapid and fatal decrease of body weight (**Fig. 1A, B and C**, **Table S1 and S2**). Of notice, moribund mice were sacrified before termination of the experiments as described in the Material and Methods. Within the susceptible mice, A/J were significantly different compared to DBA/2J and 129S2/SvPasCRL showing a faster decrease of body weight at day one (**Table S3**) and a kinetic of death significantly more rapid (**Table S1**). Resistant mice BALB/cAnNCrl and C3H/HeOuJ showed a significant lower susceptibility to *P. aeruginosa* infection compared with A/J, DBA/2J and 129S2/SvPasCRL with cases of survival, a mean survival time of at least three days and a progressive weight recovery of the survivors at day seven (**Fig. 1A, B and C** **, Table S1–S3**). Within the resistant mice, BALB/cAnNCrl and C3H/HeOuJ were not significantly different for the body weight changes, kinetic of death and survival time. The above described differences in resistance and susceptibility of most deviant inbred mice were confirmed by infecting with a lower *P. aeruginosa* infection dose of 5×10^5^ CFU.(**Figure S1 and Table S4–S6**).

### Impaired cell-mediated immunity leads to faster replication of *P. aeruginosa* in A/J mice when compared to C3H/HeOuJ

**Figure 2:**
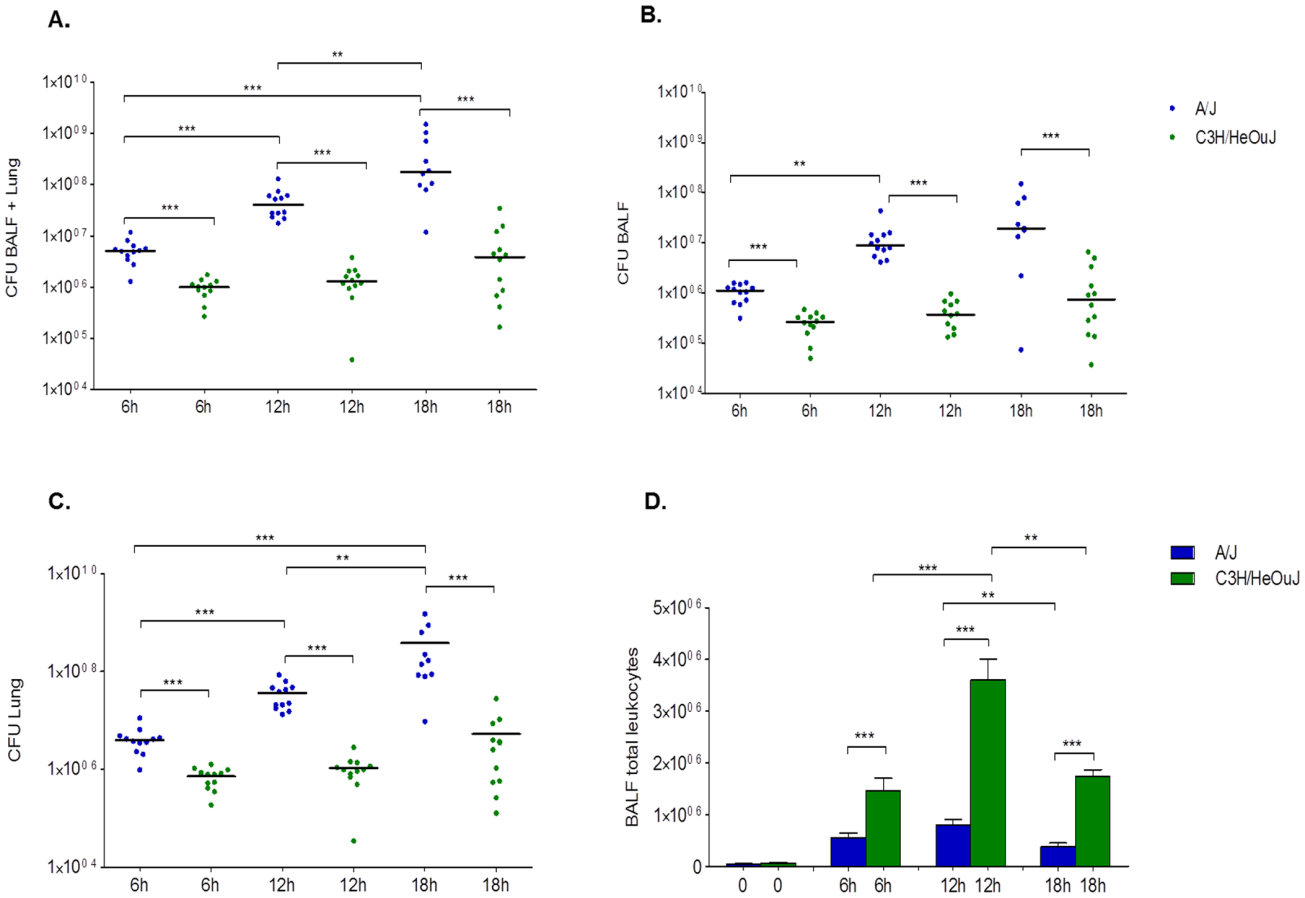
*P. aeruginosa* load and leukocytes recruitment in BALF and lung of A/J and C3H/HeOuJ mice. A/J (n = 12, for each time) and C3H/HeOuJ (n = 12, for each time) mice were challenged with 5×10^6^ CFU of AA2 clinical stain and analysed during a time course post-infection. Bacterial loads in the BALF+Lung (**A**), BALF (**B**) and lung (**C**) and were counted at 6, 12 and 18 hours in surviving mice. Dots represent CFUs in individual mice and horizontal lines represent median values reported in log scale. Total leukocytes were analyzed in BALF of *P. aeruginosa* infected mice (**D**). Bars represent median values and the error bars indicate the standard error of the mean (SEM). Blue is referred to A/J and green to C3H/HeOuJ. The data are pooled from two independent experiments. Statistical significance by Mann-Whitney U test is indicated: **p<0.01, ***p<0.001, ****p<0.0001.

Next, one of the most susceptible and resistant mouse strains were characterized for their deviant clinical and immunological phenotypes after *P. aeruginosa* AA2–induced acute pneumonia. The *P. aeruginosa* load and immune response of infected mice in terms of leukocyte recruitment, myeloperoxidase activity, and local cytokine production in the airways were investigated in A/J and C3H/HeOuJ mice during an early time course (6, 12 and 18 hours post-infection). Starting from a challenge of 5×10^6^ CFU, significant increase of total bacterial load up to 2 log_10_ CFU (4.1×10^8^) at 18 hours in the lung of susceptible A/J mice was observed indicating an uncontrolled replication of bacterial cells (**Fig. 2A**). A/J mice showed signs of bacteremia with systemic dissemination of bacterial cells in the spleen as indicated by high CFUs at 24 h post infection in moribund mice (data not shown). Conversely, at the same time points, the bacterial load in the lungs of resistant C3H/HeOuJ mice was unchanged in respect to the initial inoculum (6.9×10^6^) suggesting that resistant mice are able to keep in check the infection. A/J susceptible and C3H/HeOuJ resistant mice were significantly different in their bacterial load at all time points. Similar differences were also observed in different airways compartments as assessed by the Broncho Alveolar Lavage Fluid (BALF) (**Fig. 2B**) and lung analysis (**Fig. 2C**).

**Figure 3:**
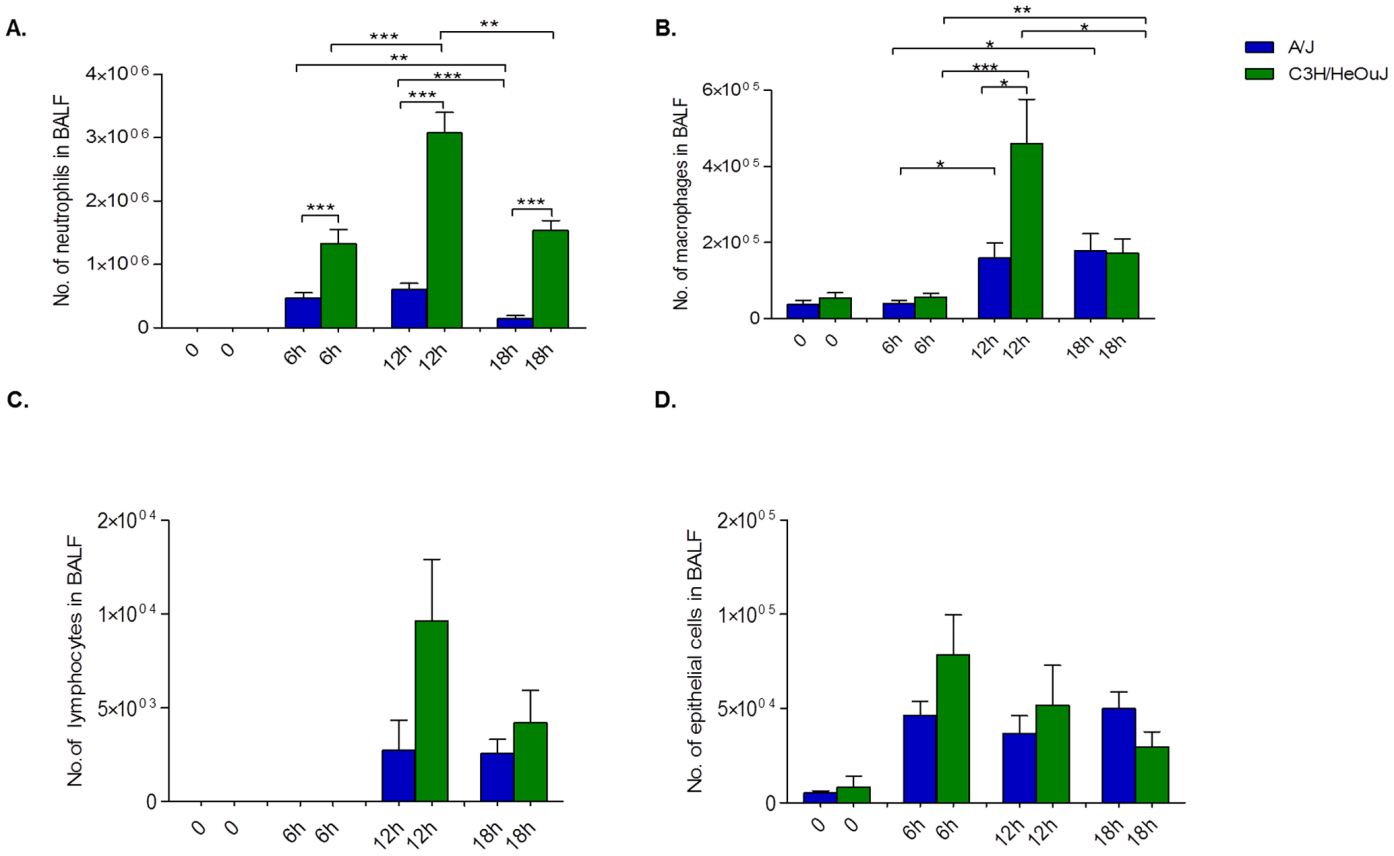
Lung inflammatory response in susceptible A/J and resistant C3H/HeOuJ *P*. *aeruginosa*-infected mice. The number of neutrophils (**A**), macrophages (**B**), lymphocytes (**C**) and epithelial cells (**D**) recruited in the airways were determined in the BALF from A/J (n = 12) (blue bar) and C3H/HeOuJ (n = 12) (green bar) mice by cytospin after 6, 12 and 18 hours of *P. aeruginosa* infection with 5×10^6^ CFU of AA2 clinical isolate. Bars represent mean values and the error bars the standard error of the mean (SEM). The data are pooled from two independent experiments. Statistical significance by Mann-Whitney U test is indicated: *p<0.05, **p<0.01, ***p<0.001.

Leukocytes recruitment in the BALF of susceptible A/J mice early after *P. aeruginosa* infection was significantly lower than C3H/HeOuJ resistant mice (**Fig. 2D**). Furthermore, while the low leukocytes numbers remained stable in A/J during 18 hours, their recruitment in C3H/HeOuJ increased and reached the peak at 12 h. In particular, a significant increase in neutrophil levels for C3H/HeOuJ, compared to A/J was observed during 18 h (**Fig. 3A**). The higher level of myeloperoxidase (MPO) activity in the BALF of C3H/HeOuJ compared to A/J supported these data (**Fig. S2**). Macrophages were also significantly higher in the lung of C3H/HeOuJ mice compared to A/J mice at 12 hours post infection, but no striking differences were present at 6 and 18 hours post infection (**Fig. 3B**). Lymphocytes and epithelial cells showed a trend being higher in C3H/HeOuJ mice but did not reach significance (**Fig. 3C and D**).

**Figure 4:**
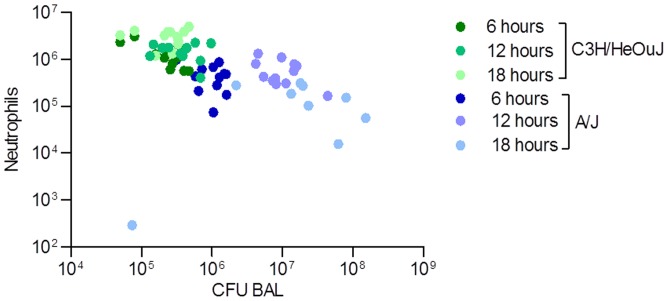
Correlation of CFU/neutrophils in the BAL of susceptible A/J and resistant C3H/HeOuJ *P*. *aeruginosa*-infected mice. CFUs and neutrophils recovered in the BAL were plotted for the two murine strains during 18 hours of *P. aeruginosa* infection. Dots represent CFUs and neutrophils in individual mice. Blue dots represent A/J mice (n = 12, for 6 and 12 hours, n = 9 for 18 hours) and green dots C3H/HeOuJ mice (n = 12 for each time). The data are pooled from two independent experiment.

When CFUs and neutrophils recovered in the BAL were plotted together, a distinct trend was observed in A/J and C3H/HeOuJ mice during 18 hours of infection. **Fig 4** showed a lower number of neutrophils recruited and a higher number of *P. aeruginosa* CFUs in A/J mice in comparison to C3H/HeOuJ mice. In particular, looking at the ratio of *P*. *aeruginosa* CFUs and neutrophils, significant differences were found (**Fig S3**). During the whole time-course, the ratio between CFUs and neutrophils was significantly lower for C3H/HeOuJ mice when compared to A/J mice, indicating a higher capacity of C3H/HeOuJ mice to control *P. aeruginosa* infection.

### A/J mice showed higher cytokines and chemokines profile in the airways when compared to C3H/HeOuJ mice during acute *P. aeruginosa* pneumonia

To better characterize the airway inflammatory response of the two deviant inbred mouse strains, we measured the concentration of a large panel of twenty-three cytokines and chemokines in murine lung homogenates. As shown in **Table 1**, the overall levels of pro-inflammatory cytokines were significantly higher in the lung of A/J mice respect to C3H/HeOuJ mice and reach significance for IL-17, G-CSF, GM-CSF, IFN-γ, IL-6, IL-1α, KC, MCP1 and MP1 in at least one time point. Other cytokines, TNF-α and IL-5, were higher in A/J compared to C3H/HeOuJ mice but did not reach significance. Thus, A/J mice showed an excessive release of pro-inflammatory cytokines that does not correlate with cellular response when compared to C3H/HeOuJ mice.

**Table 1:** Cytokines and chemokines levels in lung homogenates of susceptible A/J and resistant C3H/HeOuJ mice infected with *P*. *aeruginosa* during a time course.

	A/J			C3H/HeOuJ			A/J vs C3H/HeOuJ		
Cytokines	6 h	12 h	18 h	6 h	12 h	18 h	6 h	12 h	18 h
IL1α	777.405	443.0235	157.775	378.485	489.875	241.995	*	ns	*
IL1β	726.39	1151.41	393.915	462.985	492.23	302.1	ns	ns	ns
IL2	nd	nd	nd	nd	nd	nd	-	-	-
IL3	3.485	3.465	1.785	1.505	1.835	1.385	ns	*	ns
IL-4	15.595	13.555	12.085	9.675	12.67	10.725	ns	ns	ns
IL5	8.62	7.42	5.165	4.205	4.05	5.675	ns	ns	ns
IL6	473.33	378.19	193.035	305.56	201.27	176.795	ns	*	ns
IL9	35.7	36.43	48.495	35.705	35.7	55.195	ns	ns	ns
IL10	39.25	49.095	32.04	20.44	30.75	23.51	*	*	ns
IL12p40	56.4	21.655	23.855	14.59	19.8	21.21	*	ns	ns
IL-12p70	254.15	182.17	113.995	164.805	228.87	147.245	ns	ns	ns
IL13	71.26	74.4	73.515	40.14	55.46	65.09	*	*	ns
IL17	18.945	20.26	22.41	9.825	12.41	14.525	*	*	ns
eotaxin	nd	nd	nd	nd	nd	nd	-	-	-
G CSF	1778.86	5840.23	6713.8	607.365	2067.78	3266.77	*	*	ns
GM CSF	170.26	156.01	151.21	124.38	129.5	123.595	ns	*	ns
IFNγ	8.43	6.69	1.97	2.545	7.55	2.105	*	ns	ns
KC	68190	69190	69290	5793.96	6747.85	3068.26	*	ns	ns
MCP1	2826.25	1752.27	1109.07	757.01	1503.73	731.82	*	ns	ns
MIP1α	963.815	711.72	577.235	270.05	487.66	365.4	*	ns	ns
MIP1β	62.63	69.04	58.95	42.735	44.59	37.83	*	ns	ns
RANTES	32.67	45.24	27.125	15.22	25.115	34.03	ns	ns	ns
TNFα	18.08	15.105	16.6	9.76	12.38	15.105	ns	ns	ns

Data are expressed as median of pg/500 ug lung.

Statistical analysis for comparison of A/J vs C3H/HeOuJ at each time point by the non-parametric Mann-Whitney U test (*p<0.05) is reported.

Nd: not detectable; ns: not significant.

### Pathological differences in A/J and C3H/HeOuJ mice during *P. aeruginosa* acute pneumonia

**Figure 5:**
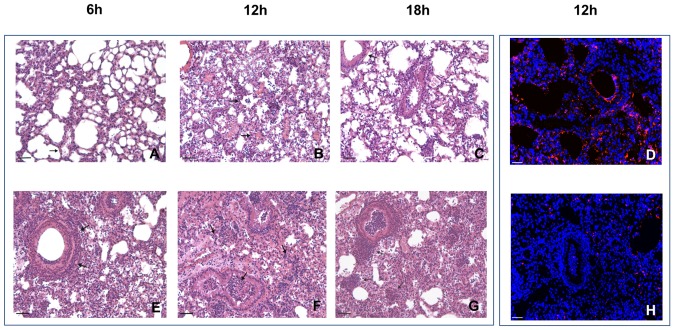
Histopathology in susceptible A/J and resistant C3H/HeOuJ *P*. *aeruginosa*-infected mice. The lungs of A/J (**A–D**) and C3H/HeOuJ (**E–H**) were stained with H&E (**A–C, E–H**) and in immunofluorescence with specific antibody against *P. aeruginosa* (red) (**D, H**). Counterstaining was performed with 49,6-Diamidino-2-phenylindole dihydrochloride (DAPI) (blue). After time course analysis, extensive infection and inflammation were visible in murine lungs with major differences between A/J and C3H/HeOuJ. Arrows indicate aggregates of lynphocytes infiltrates. Bars, 200 µm for H&E images, 100 µm for immunofluorescence images. Severity of lesions and lung involvement was scored as reported in **Fig S4**.

The histopathologic analysis of *P. aeruginosa* AA2–induced acute pneumonia revealed striking differences between A/J and C3H/HeOuJ mice (**Fig. 5** and **Fig. S4**). During an early time course a fast and consistent recruitment of inflammatory cells in C3H/HeOuJ mice compared to a delayed and lower recruitment in A/J was observed (**Fig. 5** **A-C, E-G**).

More in detail, during early phases of infection pathologic scores showed that the high inflammatory response of C3H/HeOuJ resistant mice was characterized by strong polymorphonuclear leukocytes (PMNs) recruitment in BALF and in the interstitial areas of the lungs followed by a progressive increase in macrophages involvement and a moderate expansion of aggregates of lymphocytic infiltrates (**Fig. 5** **, E–G** and **Fig. S4**). These cell-mediated response may have a key role in mounting an effective response to control bacterial clearance. Indeed, immunofluorescence staining showed low number of *P. aeruginosa* cells in the lungs of C3H/HeOuJ mice (**Fig. 5H**).

The susceptible A/J strain responded in a delayed manner characterized by a lower cell recruitment associated to low damage in the lung, and absence of a relevant interstitial/BALF response (**Fig. 5** **A–C** and **Fig. S4**). The aggregates of lymphocytic infiltrates observed in C3H/HeOuJ mice were substantially never observed in A/J susceptible strain. Indeed, immunofluorescence staining showed high numbers of *P. aeruginosa* cells localized both within the bronchial lumen and in the alveolar space indicating inadequate response of A/J mice to control bacterial replication in the lung (**Fig. 5D**).

## Discussion

An increasing number of scientific reports that the host genetic background significantly modulates the outcome of infectious diseases highlighting that in most cases multiple complex genetic interactions may have a key role in controlling infection [10]. These findings reveal that pathogens and their associated phenotypes are not the only determinants of the corresponding infectious diseases. In the case of *P. aeruginosa* opportunistic infection, much influence on disease outcome has been mainly attributed to different bacterial phenotypes rather than host genetic background. Here, we selected nine inbred murine strains characterized by a wealth of genetic and phenotypic diversity and representing a discrete part of the numerous hallmarks of the human population [14]. We show that different inbred murine strains are highly variable in their response to acute airway infection. During a time course analysis, a wide-range response to *P. aeruginosa* infection has been observed both in the survival rate and body weight change of different inbred murine strains. Most notably, deviant clinical phenotypes were observed being the A/J, 129S2/SvPasCRL and DBA/2J as the most susceptible while BALB/cAnNCrl and C3H/HeOuJ the most resistant murine strains. Other murine strains (BALB/cJ, BALB/cByJ, C57BL/6J and C57BL/6NCrl) showed intermediate phenotype. Furthermore, we demonstrate no significant behavioral differences among distinct sub-strains of C57BL/6 from Jackson (C57BL/6J) or Charles River (C57BL/6 NCrl). As reported in other studies, recent observations suggest that different variants of C57BL/6 mice are similar when monitored for acute mortality but have a different susceptibility to *P. aeruginosa* infection in terms of chronic persistence [5]. However, when distinct sub-strains of BALB/c from Jackson (BALB/cJ and BALB/cByJ) or Charles River (BALB/cAnNCrl) were compared, we find significant differences in terms of survival between BALB/cAnNCrl and BALB/cByJ. In this context, it is interesting to note that the widely used CB57BL/6 mice were not the most susceptible to *P. aeruginosa* infection compared to other inbred strains. It is worth mentioning, that C57BL/6J is also the background of *Cftr*-ko mice and it is known that these mice do not mimic human CF lung disease as in humans [22]. Our results open the question whether other mouse genetic background may better recapitulate the pulmonary abnormalities of CF patients during *P. aeruginosa* infection.

Some of the inbred mouse strains used in this work were tested previously by others employing different model systems of *P. aeruginosa* infection. So far, three publications directly compared more than four inbred mouse strains [19], [20], [21]. Intraperitoneal *P. aeruginosa* infection of 16 different strains of inbred mice showed enhanced resistance for C3H mice and susceptibility for A.BY [19]. Most strains, including DBA/2J, C57BL/6J and BALB/c, used also in our work, showed no significant differences. In another work, 11 inbred mouse strains were compared using aerosol model of *P. aeruginosa* infection [21]. DBA/2J were the most susceptible while A/J, C3H/HeN, SWR/J and B10.D2/nSnJ were the most resistant when monitored for mortality. Using a chronic model of *P. aeruginosa* infection and evaluating mortality and bacterial load, a more restricted number of four inbred mouse strains were compared. BALB/c mice were found to be resistant, and DBA/2J mice were identified as the most susceptible strain while C57BL/6NHsd and A/JCr mice were found to be relatively susceptible [20]. Additional publications characterized inflammatory response directly comparing one resistant and one susceptible mice considering as resistant and susceptible BALB/c and C57BL/6 [24], [25], BALB/c and DBA/2 [26], C3H/HeN and BALB/c respectively [27], [28]. Taken together all of previous reports and our results, it seems that a comprehensive classification of mouse inbred strains into categories according to their resistance or susceptibility is difficult to achieve. As demonstrated for other pathogens, the differences in the disease outcome may be affected by the site of infection [29], [30]. In the above mentioned works, different routes of administration have been used for *P. aeruginosa* infection including intraperitoneal [19], aerosol [21] or intratracheal (this work and [20]). Other factors have also been shown to influence the disease phenotype and, by interference, susceptibility or resistance. The size of the inoculum and the strain of the pathogen used seem to be particularly important. Different *P. aeruginosa* bacterial strains have been used, including laboratory PAO1 [21] or clinical strains PA-103 [19], PA-508 [20] and AA2 in this work. Furthermore, the final read-out measured in above mentioned publications (CFUs at different time point [20], [21] and mortality [19], [21] and our work) may affect the overall classification of the inbred strains. These results suggest a previously unrevealed level of complexity and show that conclusions regarding disease susceptibility induced by *P. aeruginosa* infection may be highly dependent on the experimental model that is used.

Mouse inbred strains are the starting point from which to explore causal phenotype-genotype relationships, including the identification of cell-mediate immune response and gene mapping. In this work, the susceptible A/J mice and the resistant C3H/HeOuJ have been used to gain deeper insight into the cellular and molecular factors that may contribute to different disease pathogenesis. High bacterial replication and inadequate immune-response were observed for A/J mice suggesting that susceptible mice are inefficient to keep in check the bacterial infection. In other words, A/J mice do not mount a proper, early immune defence leading to a permissive environment for bacterial replication and spreading from the airways to other organs and ultimately to a fast disease progression. The lack of the inflammatory response in A/J mice may not be attributable to deficient cytokines or chemokines response, rather mice showed an excessive or uncontrolled release of pro-inflammatory cytokines, suggesting an ongoing cytokine storm. Previous studies have demonstrated that A/J mice are known to carry a loss-of-function mutation at Hc gene encoding for hemolytic complement, which is implicated in mobilizing inflammatory cells, in particular neutrophils, critical for host defense against infection [31]. This deficiency has been widely demonstrated to account for the susceptibility of A/J mice to several pathogens, such as *Candida albicans* [32] and *Mycobacterium tuberculosis* [33]. However, deficiency of Hc gene may not be completely responsible for *P. aeruginosa* susceptibility since inbred strains deficient (A/J and DBA/2) or sufficient for Hc gene (129S2/SvPasCRL) are equally susceptible to *P. aeruginosa* infection.

In contrast to susceptible A/J mice, resistant C3H/HeOuJ mice mount a faster and consistent immune-response that is able to efficiently control bacterial replication. In more detail, C3H/HeOuJ mice exhibited a prompter recruitment of inflammatory cells, mainly PMNs, to the site of infection than did mice of the A/J strain. A balanced level of cytokines or chemokines has been observed in C3H/HeOuJ mice. Furthermore, after a strong neutrophilic response of C3H/HeOuJ resistant mice, a progressive increase in macrophages involvement and aggregates of lymphocytic infiltrates was observed. These features, instead, were substantially never observed in A/J susceptible strain. These differences clearly indicates that, in this model of infection, the two mouse strains react differently in modulation of inflammatory response and probably in the way of the antigen presentation to the lymphocytes. These cells may play a role in mounting a cell-mediated response to determine effective control or bacterial clearance. Of notice, after longer time from *P. aeruginosa* challenge (7 days) C3H/HeOuJ resistant mice cleared infection and resolved inflammation, with no sign of tissue damage (data not shown).

Other reports on inbred mice analysed the inflammatory response in susceptible and resistant strains showing controversial results about the biological significance of the early inflammatory response in modulating the course and outcome of *P. aeruginosa* infection. In agreement with our results, Morissette and co-workers reported a rapid influx of PMN which was shortly followed by an efficient clearance of bacteria in BALB/c resistant mice, while DBA/2 susceptible mice had a delay in both the inflammatory response and the initiation of bacterial clearance [20], [26]. However, other papers report an accumulation of inflammatory cells in susceptible rather than resistant mice. An exaggerated inflammatory response dominated by PMNs correlates with susceptibility to infection in C57BL/6 mice, whilst a modest inflammatory response dominated with macrophages correlated with resistance in BALB/c mice [24], [25]. Nevertheless, it should be expected that differences in the experimental model used (acute vs chronic) may affect several physiological parameters, adding complexity to the overall picture of the *P. aeruginosa*/host interaction.

Taken together, our results showed that survival to *P. aeruginosa* infection is clearly affected by host genetic background. Comparative analysis of the cell-mediated immunity to *P. aeruginosa* infection in resistant and susceptible strain has been used in determining key player of a successful versus an unsuccessful response to infection. During this early phase of infection, a prompt inflammatory response in the airways provides a biological advantage in creating a non-permissive environment for *P. aeruginosa* replication and locking the spread to other organs. Thus, we speculate that host gene(s) may have a role in the reduction of cell-mediated immunity playing a critical part in the control of *P. aeruginosa* infection. With the use of recombinant inbred strategies, the survival differences between A/J and C3H/HeOuJ mice will permit future mapping of key genes involved in *P. aeruginosa* infection.

## Materials and Methods

### Ethic Statement

Animal studies were conducted according to protocols approved by San Raffaele Scientific Institute (Milan, Italy) Institutional Animal Care and Use Committee (IACUC) and adhered strictly to the Italian Ministry of Health guidelines for the use and care of experimental animals (Permit number: 502). Research on the bacterial isolates from the individual with CF has been approved by the responsible physician at the CF center at Hannover Medical School, Germany. Patient gave informed consent before the sample collection. Approval for storing of biological materials was obtained by the Hannover Medical School, Germany.

### Bacterial strain

*P. aeruginosa* clinical isolate AA2 was obtained from a CF patient attending the Medizinische Hochschule of Hannover, Germany at the onset of chronic colonization and described before [5], [23]. The strain was cultured in trypticase soy broth (TSB) and plated on trypticase soy agar (TSA).

### Mouse model of *P. aeruginosa* acute infection

Nine inbred immune-competent male mouse strains eight weeks old namely A/J, BALB/cJ, BALB/cByJ, C3H/HeOuJ, C57BL/6J and DBA/2J were purchased from Jackson (J) and BALB/cAnNCrl, C57BL/6NCrl, 129S2/SvPasCRL from Charles River laboratories (Crl). Mice were infected with the doses of 5×10^5^ and 5×10^6^ CFU of planktonic *P. aeruginosa* clinical isolate AA2 according with established procedures [23]. Briefly, mice were anesthetized by an intra-peritoneal injection of a solution of 2.5% Avertin (2,2,2-tribromethanol, 97%; Sigma Aldrich) administered in a volume of 0.015 mlg^−1^ body weight and the trachea directly visualized by a ventral midline incision, exposed and intubated with a sterile, flexible 22-g cannula attached to a 1 ml syringe. A 50 µl inoculum of 5×10^5^ or 5×10^6^ CFU were implanted via the cannula into the lung, with both lobes inoculated. After infection, mortality and body weight were monitored over one week. Animals were observed twice a day and those showing more than 25% of body weight loss and had evidence of severe clinical disease, such as scruffy coat, inactivity, loss of appetite, poor locomotion, or painful posture, were sacrificed before the termination of the experiments with an overdose of carbon dioxide. For the time course, additional groups of mice were sacrificed by CO_2_ administration at 6, 12 and 18 hours and analysed for CFU in the lung, BALF cell count and myeloperoxidase (MPO) activity as previously described [34],[35]. In another group of mice, the lungs were excised and used for histopathology.

### BALF and lung collection and analysis

BALF was extracted with a 22-gauge venous catheter by washing the lungs with RPMI-1640 (Euroclone) with protease inhibitors (Complete tablets, Roche Diagnostic and PMSF, Sigma) [34]. Total cells present in the BALF were counted, and a differential cell count was performed on cytospins stained with Diff Quick (Dade, Biomap, Italy). BALF was serially diluted and plated on TSB-agar plates. After erythrocyte lysis with ACK lysis solution (pH 7.2; NH_4_Cl 0.15 M, KHCO_3_ 10 mM, EDTA 0.1 mM), cells were resuspended in cetyltrimethylammonium chloride 0.5% (Sigma Aldrich) and centrifuged. The clear extracts were used to analyse the MPO activity: samples were mixed with equal volumes of 3,3′,5,5′-tetramethyl-benzidine dihydrochloride for 2 min and the reaction stopped by adding H_2_SO_4_. The OD was measured at 450 nm.

### Lung homogenization and cytokine analysis

Lungs were removed and homogenized in PBS with Ca2+/Mg2+ containing protease inhibitors. Samples were serially diluted and plated on the above agar media for CFU counts. Lung homogenates were then centrifuged at 14,000 rpm for 30 minutes at 4°C, and the supernatants were stored at −20°C for cytokine analysis.

Total lung homogenates protein content was quantified with Bradford's assay (Bio-RAD) at the final concentration of 500 µg/ml. A panel of 23 murine chemokines and cytokines were measured using Bio-Plex pro Mouse Cytokine Standard 23-Plex, Group I according to the manufacterer's instructions.

### Histologic and immunofluorescence analysis

Lungs were removed, fixed in 10% buffered formalin for at least 24 h and embedded in paraffin. Consecutive 2-mm sections from the middle of the five lung lobes were used for histological and immunofluorescence examination in each mouse. Sections for histological analysis were stained by Haematoxylin-Eosin according to standard procedures. To grade inflammation severity and extent (e.g diffuse, intraluminal and/or interstitial), alveolar damage, bronchial involvement, and percentage of parenchyma involved histological score analysis of murine lungs was performed as described in Methods S1.

Localization of *P. aeruginosa* was performed in de-paraffinized lung sections by employing a rabbit antiserum specific for *P*. *aeruginosa* and Texas Red-labelled goat anti-rabbit IgG as described [5]. The slides were examined using an Axioplan fluorescence microscope (Zeiss) and images were taken with a KS 300 imaging (Kontron).

### Statistical analysis

Results are presented as median or mean ±SEM. Because of the small sample size and the non-normal distribution of variables, we used a nonparametric approach for most part of analysis. The Mann –Whitney U test was used to compare CFU, cells, MPO activity, cytokines levels and histologic measurements between A/J and C3H/HeOuJ mice. Mantel–Cox test was used to determine the significance of differences in means between pairs. Two- way ANOVA with Bonferroni's Multiple Comparison test was used to compare change in body weight. One-way ANOVA with Bonferroni's Multiple Comparison test was used to compare the mean survival time. Tests are considered statistically significant when the significance level is ≤0.05.

## Supporting Information

Figure S1 **Survival, body weight and mean survival time after** ***P. aeruginosa*** **infection in inbred mouse strains.**
(TIF)

Figure S2 **Myeloperoxidase activity in susceptible A/J and resistant C3H/HeOuJ** ***P*** **.** ***aeruginosa*** **-infected mice.**
(TIF)

Figure S3 **Ratio CFU/neutrophils in the BALF of susceptible A/J and resistant C3H/HeOuJ** ***P*** **.** ***aeruginosa*** **-infected mice.**
(TIF)

Figure S4 **Histopathological scores in susceptible A/J and resistant C3H/HeOuJ** ***P*** **.** ***aeruginosa*** **-infected mice.**
(TIF)

Table S1 **Statistical comparison of survival between inbred mice infected with 5×10^6^** ***P*** **.** ***aeruginosa*** **.**
(DOC)

Table S2 **Statistical comparison of Mean Survival Time between inbred mice infected with 5×10^6^** ***P*** **.** ***aeruginosa.***
(DOC)

Table S3 **Statistical comparison of change in body weight between inbred mice infected with 5×10^6^** ***P*** **.** ***aeruginosa*** **.**
(DOC)

Table S4 **Statistical comparison of survival between inbred mice infected with 5×10^5^** ***P*** **.** ***aeruginosa.***
(DOC)

Table S5 **Statistical comparison of Mean Survival time between inbred mice infected with 5×10^5^** ***P*** **.** ***aeruginosa***
(DOC)

Table S6 **Statistical comparison of change in body weight between inbred mice infected with 5×10^5^** ***P*** **.** ***aeruginosa*** **.**
(DOC)

Methods S1 (DOC)
