# Supplementary figures and images for: Host Genetic Background Influences the Response to the Opportunistic Pseudomonas aeruginosa Infection Altering Cell-Mediated Immunity and Bacterial Replication
Source: PLoS One. 2014 Sep 30;9(9):e106873. doi: 10.1371/journal.pone.0106873 (PMC4182038; doi:10.1371/journal.pone.0106873)

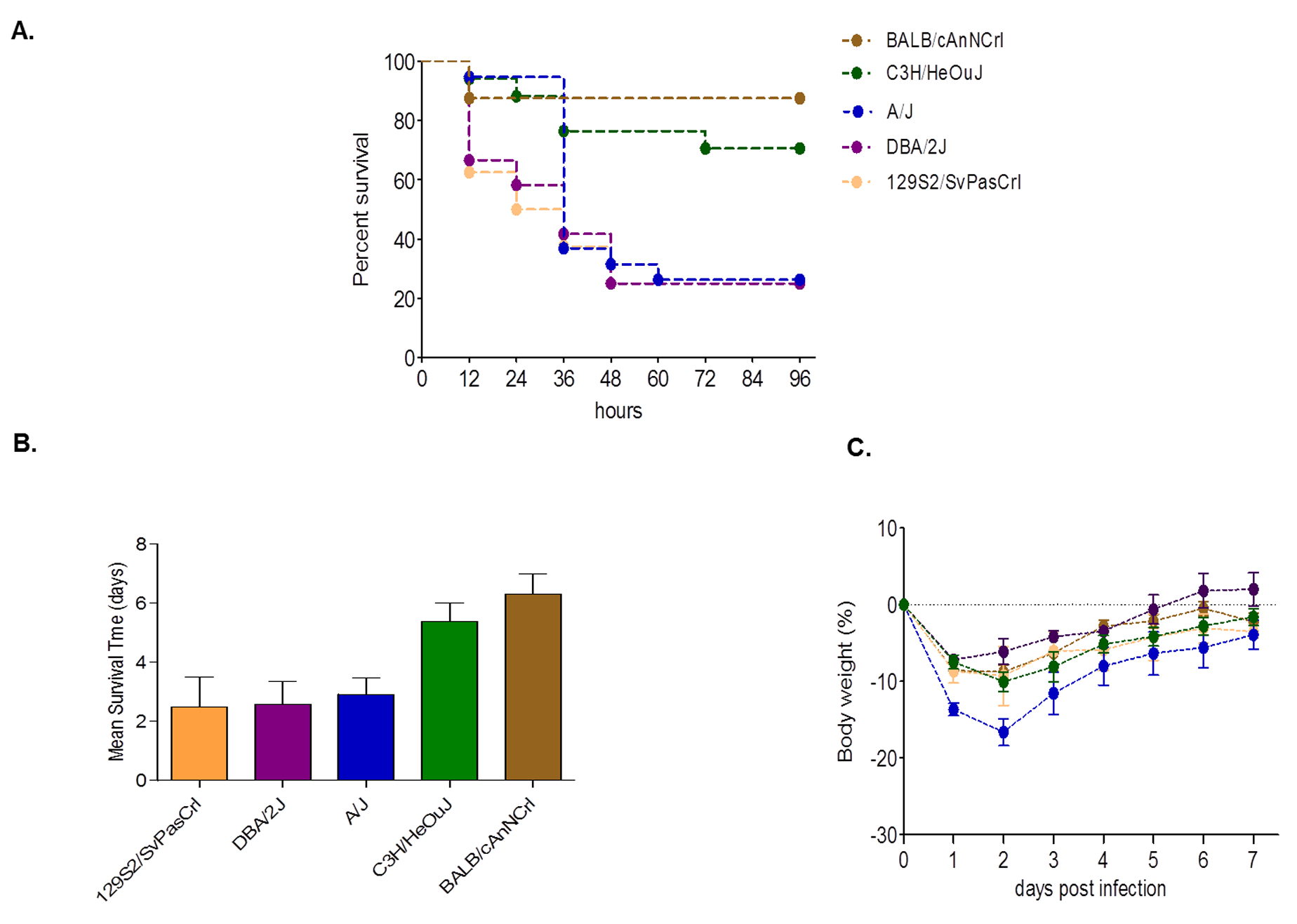

Supplement: Figure S1 — Survival, body weight and mean survival time after P. aeruginosa infection in inbred mouse strains. (TIF) [file pone.0106873.s001.tif]

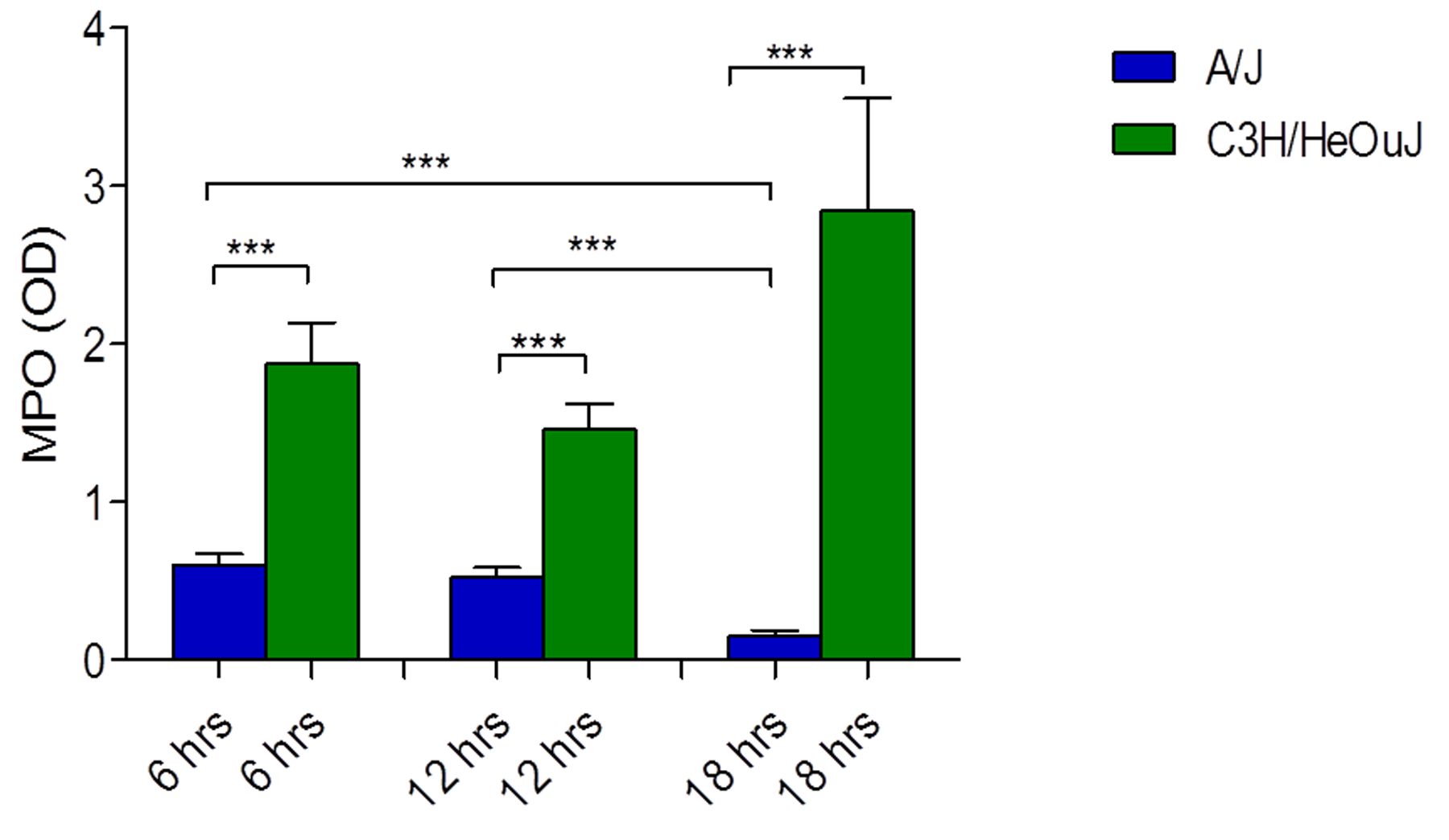

Supplement: Figure S2 — Myeloperoxidase activity in susceptible A/J and resistant C3H/HeOuJ P . aeruginosa -infected mice. (TIF) [file pone.0106873.s002.tif]

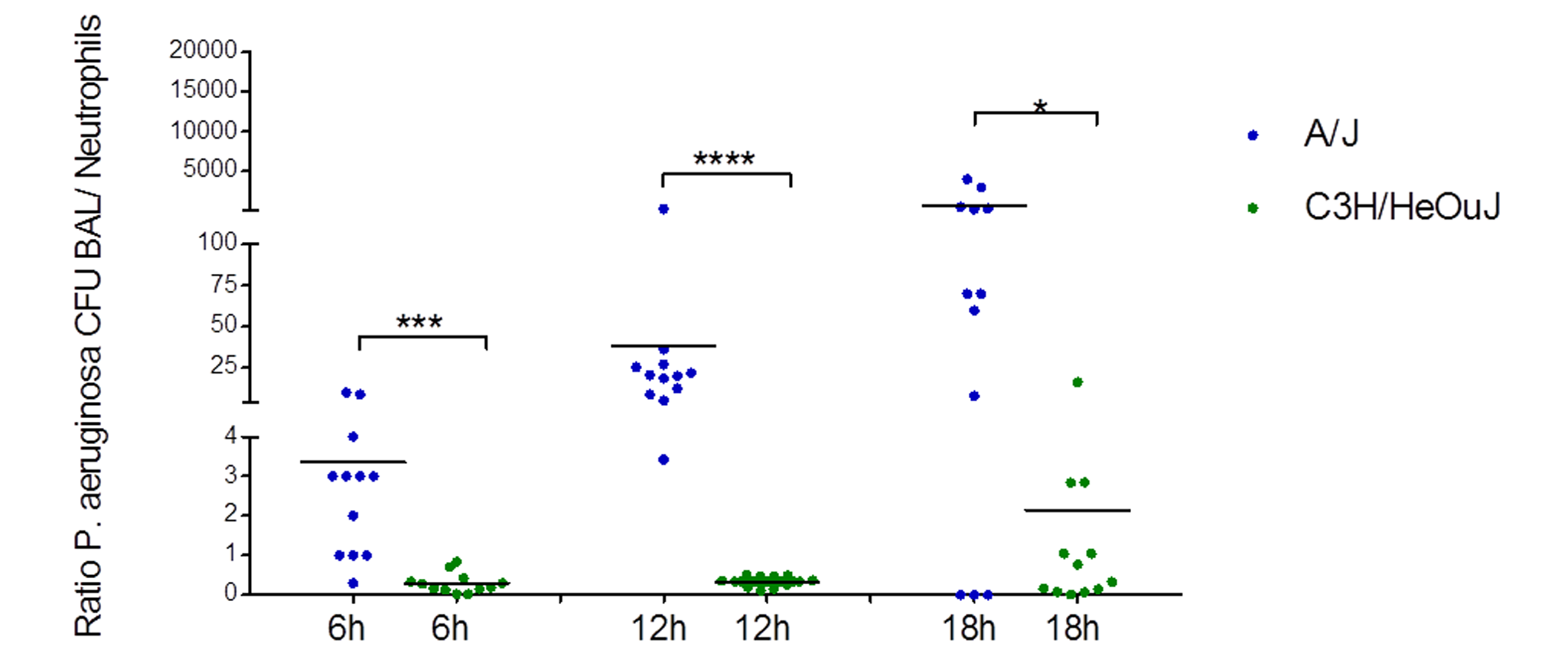

Supplement: Figure S3 — Ratio CFU/neutrophils in the BALF of susceptible A/J and resistant C3H/HeOuJ P . aeruginosa -infected mice. (TIF) [file pone.0106873.s003.tif]

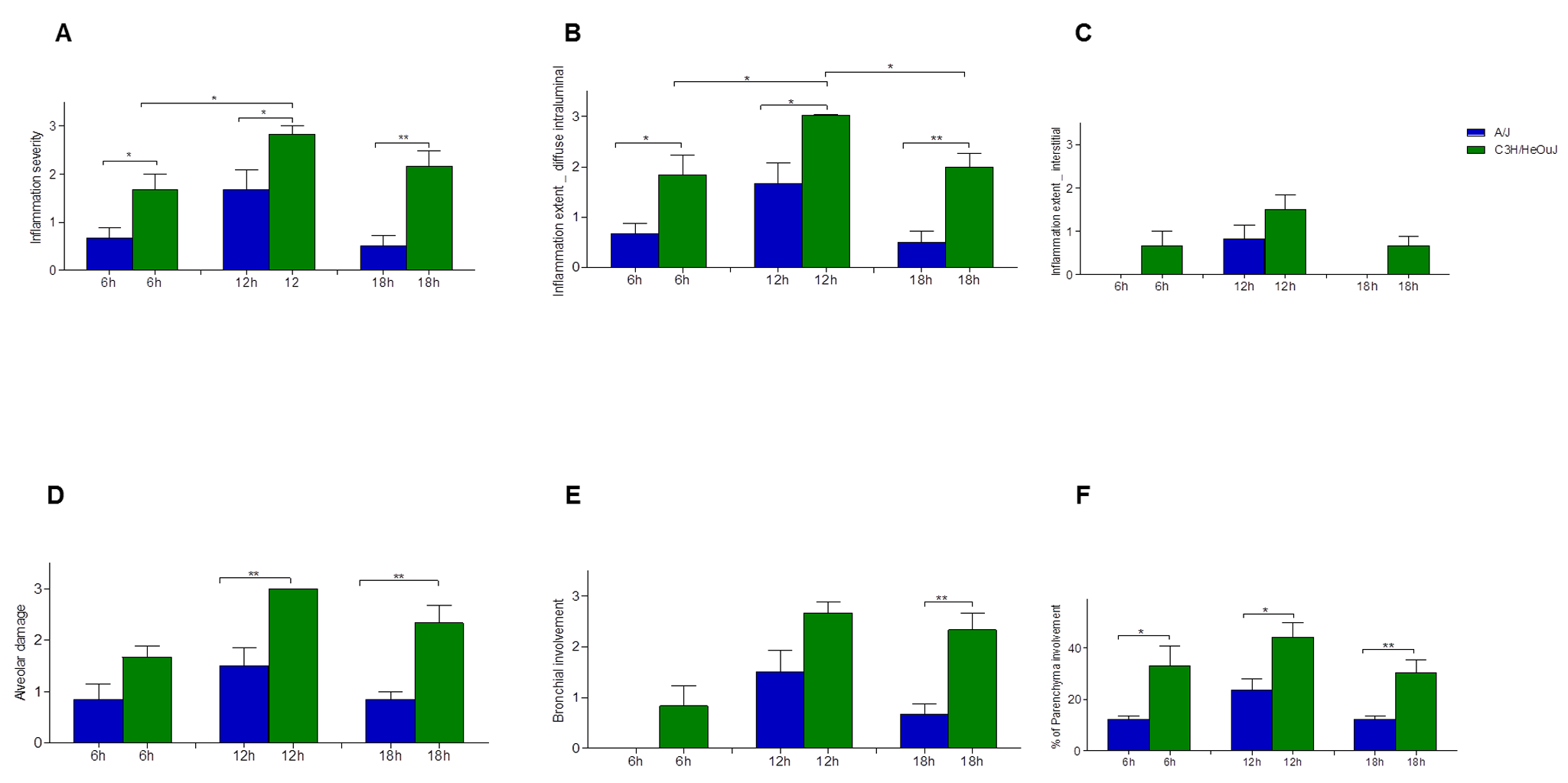

Supplement: Figure S4 — Histopathological scores in susceptible A/J and resistant C3H/HeOuJ P . aeruginosa -infected mice. (TIF) [file pone.0106873.s004.tif]
