# Supplementary material for: Host Genetic Background Influences the Response to the Opportunistic Pseudomonas aeruginosa Infection Altering Cell-Mediated Immunity and Bacterial Replication
Source: PLoS One. 2014 Sep 30;9(9):e106873. doi: 10.1371/journal.pone.0106873 (PMC4182038; doi:10.1371/journal.pone.0106873)
Supplement: Table S1 — Statistical comparison of survival between inbred mice infected with 5×10^6^ P . aeruginosa . (DOC) [file pone.0106873.s005.doc]

**Table S1. Statistical comparison of survival between inbred mice infected with 5x10^6^ *P*. *aeruginosa*.**

| **Strain** | | **129S2/SvPasCrl** | **DBA/2J** | **BALB/cJ** | **C57BL/6J** | | **BALB/cByJ** | **C57BL/6NCrl** | **C3H/HeOuJ** | **BALB/cAnCrl** |
| --- | --- | --- | --- | --- | --- | --- | --- | --- | --- | --- |
| **A/J** | * | | ** | ** | | *** | ** | **** | **** | **** |
| **129S2/SvPasCrl** |  | | ns | ns | | * | ns | ** | *** | *** |
| **DBA/2J** |  | |  | ns | | ns | ns | ns | ** | ** |
| **BALB/cJ** |  | |  |  | | ns | ns | ns | ns | ns |
| **C57BL/6J** |  | |  |  | |  | ns | ns | ns | ns |
| **BALB/cByJ** |  | |  |  | |  |  | * | * | * |
| **C57BL/6NCrl** |  | |  |  | |  |  |  | ns | ns |
| **C3H/HeOuJ** |  | |  |  | |  |  |  |  | ns |

Statistical significance by Mantel-Cox test for survival was performed among the nine inbred mouse strains (*p<0.05, **p<0.01, ***p<0.001****, p<0.0001, ns not significant)
