# Supplementary material for: Host Genetic Background Influences the Response to the Opportunistic Pseudomonas aeruginosa Infection Altering Cell-Mediated Immunity and Bacterial Replication
Source: PLoS One. 2014 Sep 30;9(9):e106873. doi: 10.1371/journal.pone.0106873 (PMC4182038; doi:10.1371/journal.pone.0106873)
Supplement: Table S2 — Statistical comparison of Mean Survival Time between inbred mice infected with 5×10^6^ P . aeruginosa. (DOC) [file pone.0106873.s006.doc]

**Table S2. Statistical comparison of Mean Survival Time between inbred mice infected with 5x10^6^ *P*. *aeruginosa.***

| **Strain** | **129S2/SvPasCrl** | **DBA/2J** | | | **BALB/cJ** | **C57BL/6J** | | **BALB/cByJ** | | **C57BL/6NCrl** | **C3H/HeOuJ** | **BALB/cAnCrl** | |
| --- | --- | --- | --- | --- | --- | --- | --- | --- | --- | --- | --- | --- | --- |
| **A/J** | ns | | ns | ns | | | ns | | ns | ns | * | | *** |
| **129S2/SvPasCrl** |  | | ns | ns | | | ns | | ns | ns | * | | * |
| **DBA/2J** |  | |  | ns | | | ns | | ns | ns | ns | | * |
| **BALB/cJ** |  | |  |  | | | ns | | ns | ns | ns | | ns |
| **C57BL/6J** |  | |  |  | | |  | | ns | ns | ns | | ns |
| **BALB/cByJ** |  | |  |  | | |  | |  | ns | ns | | ns |
| **C57BL/6NCrl** |  | |  |  | | |  | |  |  | ns | | ns |
| **C3H/HeOuJ** |  | |  |  | | |  | |  |  |  | | ns |

Statistical significance by One- way ANOVA with Bonferroni’s Multiple comparison test for mean survival time was performed among the nine inbred mouse strains (*p<0.05, ***p<0.001, ns not significant).
