# Supplementary material for: Host Genetic Background Influences the Response to the Opportunistic Pseudomonas aeruginosa Infection Altering Cell-Mediated Immunity and Bacterial Replication
Source: PLoS One. 2014 Sep 30;9(9):e106873. doi: 10.1371/journal.pone.0106873 (PMC4182038; doi:10.1371/journal.pone.0106873)
Supplement: Table S4 — Statistical comparison of survival between inbred mice infected with 5×10^5^ P . aeruginosa. (DOC) [file pone.0106873.s008.doc]

***Table S4. Statistical comparison of survival between inbred mice infected with 5 x 10^5^  P. aeruginosa.***

| **Strain** | **DBA/2J** | **A/J** | **C3H/HeOuJ** | **BALB/cAnCrl** |
| --- | --- | --- | --- | --- |
| **129S2/SvPasCrl** | ns | ns | * | * |
| **DBA/2J** |  | ns | * | * |
| **A/J** |  |  | * | * |
| **C3H/HeOuJ** |  |  |  | ns |

Statistical significance by Mantel-Cox test for survival was performed among the five inbred mouse strains. (*p<0.05, ns not significant)
