# Supplementary material for: Host Genetic Background Influences the Response to the Opportunistic Pseudomonas aeruginosa Infection Altering Cell-Mediated Immunity and Bacterial Replication
Source: PLoS One. 2014 Sep 30;9(9):e106873. doi: 10.1371/journal.pone.0106873 (PMC4182038; doi:10.1371/journal.pone.0106873)
Supplement: Table S5 — Statistical comparison of Mean Survival time between inbred mice infected with 5×10^5^ P . aeruginosa (DOC) [file pone.0106873.s009.doc]

**Table S5. Statistical comparison of Mean Survival time between inbred mice infected with 5x10^5^ *P*. *aeruginosa***

| **Strain** | **129S2/SvPasCrl** | **DBA/2J** | **A/J** | **C3H/HeOuJ** | **BALB/cAnCrl** |
| --- | --- | --- | --- | --- | --- |
| **129S2/SvPasCrl** |  | ns | ns | ns | * |
| **DBA/2J** |  |  | ns | * | * |
| **A/J** |  |  |  | * | * |
| **C3H/HeOuJ** |  |  |  |  | ns |

Statistical significance by One- way ANOVA with Bonferroni’s Multiple comparison test for mean survival time was performed among thefive inbred mouse strains (*p<0.05, ns not significant).
