# Supplementary material for: Host Genetic Background Influences the Response to the Opportunistic Pseudomonas aeruginosa Infection Altering Cell-Mediated Immunity and Bacterial Replication
Source: PLoS One. 2014 Sep 30;9(9):e106873. doi: 10.1371/journal.pone.0106873 (PMC4182038; doi:10.1371/journal.pone.0106873)
Supplement: Table S6 — Statistical comparison of change in body weight between inbred mice infected with 5×10^5^ P . aeruginosa . (DOC) [file pone.0106873.s010.doc]

**Table S6. Statistical comparison of change in body weight between inbred mice infected with 5x10^5^ *P*. *aeruginosa*.**

| **Day** | **Strain** | **129S2/SvPasCrl** | **DBA/2J** | **BALB/cJ** | **C57BL6/J** | **BALB/cBJ** | **C57BL/6NCrl** | **C3H/HeOuJ** | **BALB/cAnNCrl** |
| --- | --- | --- | --- | --- | --- | --- | --- | --- | --- |
| 1 | **A/J** | ns | ns | ns | ns | ns | ns | * | ns |
|  | **129S2/SvPasCrl** |  | ns | ns | ns | ns | ns | ns | ns |
|  | **DBA/2J** |  |  | ns | ns | ns | ns | ns | ns |
|  | **BALB/cJ** |  |  |  | ns | ns | ns | ns | ns |
|  | **C57BL6/J** |  |  |  |  | ns | ns | ns | ns |
|  | **BALB/cBJ** |  |  |  |  |  | ns | ns | ns |
|  | **C57BL/6NCrl** |  |  |  |  |  |  | ns | ns |
|  | **C3H/HeOuJ** |  |  |  |  |  |  |  | ns |
|  | **BALB/cAnNCrl** |  |  |  |  |  |  |  |  |
| 2 | **A/J** | ns | ns | ns | ns | ns | ns | ** | *** |
|  | **129S2/SvPasCrl** |  | *** | ns | ns | ns | ns | ns | ns |
|  | **DBA/2J** |  |  | ns | ns | ns | ns | ns | ns |
|  | **BALB/cJ** |  |  |  | ns | ns | ns | ns | ns |
|  | **C57BL6/J** |  |  |  |  | ns | ns | ns | ns |
|  | **BALB/cBJ** |  |  |  |  |  | ns | ns | ns |
|  | **C57BL/6NCrl** |  |  |  |  |  |  | ns | ns |
|  | **C3H/HeOuJ** |  |  |  |  |  |  |  | ns |

Two way ANOVA test was used for pair wise comparisons between inbred mouse strains at different days (*p<0.05, **p<0.01, ***p<0.001, ns not significant).
