# Supplementary material for: Host Genetic Background Influences the Response to the Opportunistic Pseudomonas aeruginosa Infection Altering Cell-Mediated Immunity and Bacterial Replication
Source: PLoS One. 2014 Sep 30;9(9):e106873. doi: 10.1371/journal.pone.0106873 (PMC4182038; doi:10.1371/journal.pone.0106873)
Supplement: Methods S1 — (DOC) [file pone.0106873.s011.doc]

**METHODS S1**

**Histologic analysis.**

To grade inflammation severity and extent (e.g diffuse, intraluminal and/or interstitial), alveolar damage, bronchial involvement, and percentage of parenchyma involved histological score analysis of murine lungs was performed. Histological examination primarily included the assessment of inflammation by scoring the number of inflammatory cells (mononuclear cells, such as macrophages, lymphocytes, and plasma cells, and neutrophils) at a magnification of 400. The number of inflammatory cells was evaluated by using a visual analogue scale modified for murine pulmonary specimens, as described previously [1], and results are reported as the mean for the entire specimen. When considerable variation of intensity of infiltration was evident in the same specimen, the mean for several areas was determined and the specimen was scored accordingly. Neutrophils and mononuclear cells were classified as absent (score of 0) when there were no or fewer than 19 cells per high-power field (HPF) (at a magnification of 400), mild (score of 1) for 20 to 49 cells per HPF, moderate (score of 2) for 50 to 99 cells per HPF, marked or severe (score of 3) for 100 to 200 cells or more per HPF. Percent of parenchyma involved was scored as 0 when no area of the two lungs was interested. Considering 100 the entire area of the two lung sections performed in the sagittal direction, the involvement of the parenchyma was scored as 1 when approximately the 25% of the total area was occupied by inflammatory exudate; was scored as 2 when the involved area was comprised between 26 to 50%, 3 if comprised between 51 to 75%, and 4 if between 76 to 100%.

Histological criteria for normal pulmonary characteristics included detection of no or only a few mononuclear cells per HPF and no or only a few scattered neutrophils in bronchioli and alveoli without tissue changes (no interstitial thickening or aggregates of lymphocytic infiltrates and airways free from exudate).

**REFERENCES**

1. Cersini A, Martino MC, Martini I, Rossi G, Bernardini ML (2003) Analysis of virulence and inflammatory potential of Shigella flexneri purine biosynthesis mutants. Infect Immun 71: 7002-7013.
